# The Evolution of Pleconaril: Modified *O*-Alkyl Linker Analogs Have Biological Activity towards Coxsackievirus B3 Nancy

**DOI:** 10.3390/molecules25061345

**Published:** 2020-03-16

**Authors:** Alexandrina Volobueva, Anna Egorova, Anastasia Galochkina, Sean Ekins, Vladimir Zarubaev, Vadim Makarov

**Affiliations:** 1 Saint-Petersburg Pasteur Institute, Mira str., 14, 197101 Saint Petersburg, Russia; sasha-khrupina@mail.ru (A.V.); nastyagalochkina@yandex.ru (A.G.); zarubaev@gmail.com (V.Z.); 2 Bach Institute of Biochemistry, Research Center of Biotechnology of the Russian Academy of Sciences, Leninsky prospect, 33, build. 2, 119071 Moscow, Russia; anna.p.egorova@gmail.com; 3 Collaborations Pharmaceuticals, Inc., 840 Main Campus Drive, Lab 3510, Raleigh, NC 27606, USA; sean@collaborationspharma.com

**Keywords:** coxsackievirus, coxsackievirus B3 Nancy, viral myocarditis, antivirals, pleconaril

## Abstract

Coxsackieviruses type B are one of the most common causes of mild upper respiratory and gastrointestinal illnesses. At the time of writing, there are no approved drugs for effective antiviral treatment for Coxsackieviruses type B. We used the core-structure of pleconaril, a well-known antienteroviral drug candidate, for the synthesis of novel compounds with *O*-propyl linker modifications. Some original compounds with 4 different linker patterns, such as sulfur atom, ester, amide, and piperazine, were synthesized according to five synthetic schemes. The cytotoxicity and bioactivity of 14 target compounds towards Coxsackievirus B3 Nancy were examined. Based on the results, the values of 50% cytotoxic dose (CC_50_), 50% virus-inhibiting dose (IC_50_), and selectivity index (SI) were calculated for each compound. Several of the novel synthesized derivatives exhibited a strong anti-CVB3 activity (SI > 20 to > 200). These results open up new possibilities for synthesis of further new selective anticoxsackievirus compounds.

## 1. Introduction

Enteroviruses belonging to the Picornaviridae family are a diverse group of small (30–32 nm size) icosahedral non-enveloped viruses with single-stranded non-segmented positive RNA genome with a poly(A) tail. They are able to survive in harsh environments and can cause both self-limiting infections as well as polio, hand-foot-mouth disease, and heart and central nervous system diseases [1]. Currently, the genus *Enterovirus* encompasses 15 species: Enterovirus A–L and Rhinovirus A–C. Coxsackieviruses type B (CVBs) are members of Enterovirus B species and include six serotypes (CVB1-6). CVBs are lytic viruses but persistent infection responsible for chronic inflammation within target organs can be established. CVB3 often leads to mild upper respiratory and gastrointestinal illnesses, but it can also cause myocarditis [2,3]. Viral myocarditis is usually associated with dyspnea, arrhythmia, and chest pain and can lead to acute heart failure and sudden death. Currently available treatment is supportive and focuses on the symptomatic factors of disease [4,5].

To date, there are no approved antiviral agents for effective therapy of CVB3 infections. Currently the most advanced approaches for anti-CVB drug design are focused on the search for new direct antivirals, the modification of existing antiviral compounds, and drug repurposing screening [6]. Pleconaril, a well-known antienteroviral drug candidate with the capsid-binding mechanism of action, does not cover all of the Coxsackievirus B serotypes, including the typical representative, Coxsackievirus B3 Nancy, which is explored in this article [7,8,9].

**Figure 1:**
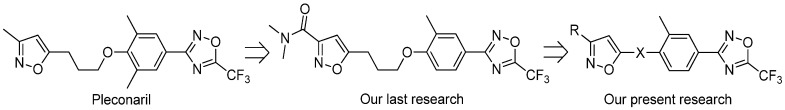
The Evolution of Pleconaril.

Previously, we have reported that pleconaril resistance was overcome by unsubstituted analogues or by monosubstitution in the central phenyl ring [10]. In our most recent work, we showed the impact of the substitution pattern in the isoxazole and phenyl rings of the pleconaril core structure and their effect on antiviral activity [11]. The most active compound to date contains the 3-*N*,*N*-dimethylcarbamoyl group in the isoxazole ring and the 3-methyl group in the phenyl ring (Figure 1).

It is interesting to investigate how the replacement of the alkyl linker with different substituents affects the antienteroviral activity, because in the original pleconaril research project, G.D. Diana et al. varied the length of the aliphatic chain only [12,13]. Thus, in the present article, we have continued our investigation to study the influence of the pleconaril core structure and various modifications on the observed antiviral activity. We synthesized compounds with the general structure shown in Figure 1, which have the *N*,*N*-dimethylcarbamoyl or ethoxycarbonyl or methyl (like pleconaril) groups in the isoxazole ring and the 3-methyl group in phenyl ring, and then examined their inhibition activity against Coxsackievirus B3 Nancy.

## 2. Results and Discussion

**Scheme 1:**
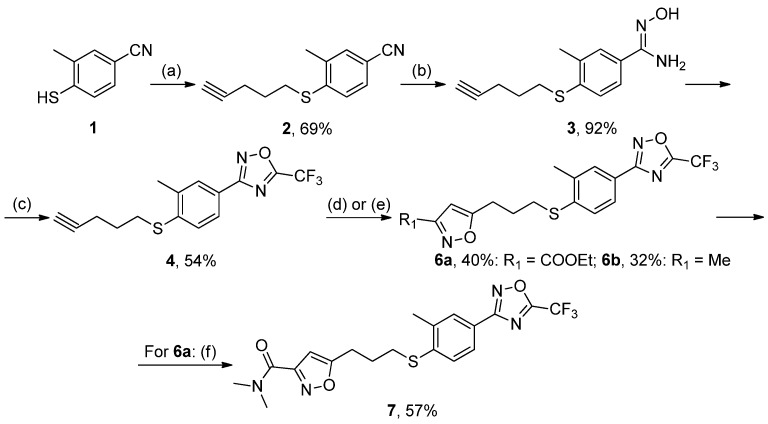
Synthesis of 3-(3-methyl-4-((3-(3-substitutedisoxazol-5-yl)propyl)thio)phenyl)-5-(trifluoromethyl)-1,2,4-oxadiazoles **6a,b** and **7**. Reagents and conditions: (a) 5-chloro-1-pentyne, K_2_CO_3_, KI, NMP, 65 °C; (b) NH_2_OH·HCl, K_2_CO_3_, EtOH_abs_, reflux; (c) (CF_3_CO)_2_O, pyridine, 80–90 °C; (d) 2-chloro-2-(hydroxyimino)acetic acid ethyl ester **5**, Et_3_N, DMF, 80–90 °C; (e) acetaldoxime, *N*-chlorosuccinimide, pyridine_cat_, Et_3_N, DMF, 80–90 °C; (f) dimethylamine solution 17 wt.% in dioxane, 70–80 °C.

**Scheme 2:**
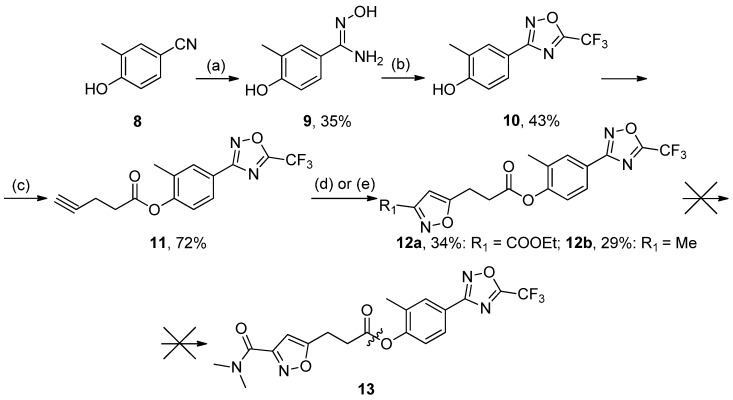
Synthesis of 2-methyl-4-(5-(trifluoromethyl)-1,2,4-oxadiazol-3-yl)phenyl ester of 3-(3-substituted isoxazol-5-yl)propionic acids **12a,b**. Reagents and conditions: (a) NH_2_OH·HCl, K_2_CO_3_, EtOH_abs_, reflux; (b) (CF_3_CO)_2_O, pyridine, 80–90 °C; (c) 4-pentynoic acid, DCC, pyridine, rt; (d) 2-chloro-2-(hydroxyimino)acetic acid ethyl ester **5**, Et_3_N, DMF, 80–90 °C; (e) acetaldoxime, *N*-chlorosuccinimide, pyridine_cat_, Et_3_N, DMF, 80–90 °C.

**Scheme 3:**
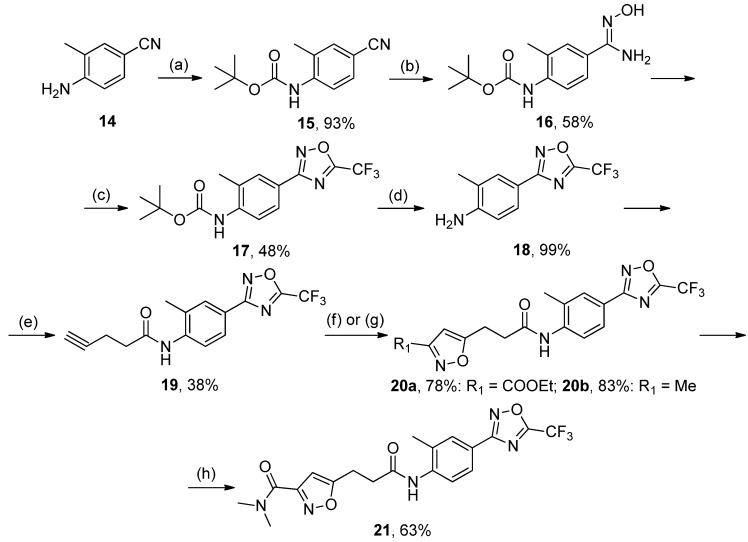
Synthesis of 2-methyl-4-(5-(trifluoromethyl)-1,2,4-oxadiazol-3-yl)phenylamide of 3-(3-substituted isoxazol-5-yl)propionic acids **20a,b** and **21**. Reagents and conditions: (a) Boc_2_O, 85 °C; (b) NH_2_OH·HCl, K_2_CO_3_, EtOH_abs_, reflux; (c) (CF_3_CO)_2_O, pyridine, 80–90 °C; (d) CF_3_COOH, DCM, rt; (e) 4-pentynoic acid, EDCl, DMAP, DCM, rt; (f) 2-chloro-2-(hydroxyimino)acetic acid ethyl ester **5**, Et_3_N, DMF, 80–90 °C; (g) acetaldoxime, *N*-chlorosuccinimide, pyridine_cat_, Et_3_N, DMF, 80–90 °C; (h) dimethylamine solution 17 wt.% in dioxane, 70–80 °C.

**Scheme 4:**
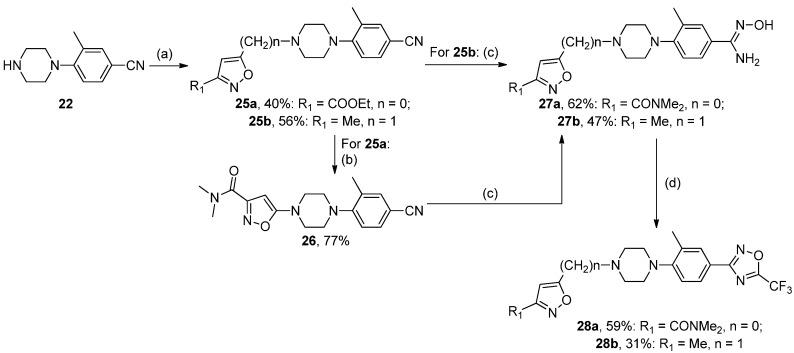
Synthesis of piperazine derivatives **28a,b**. (a) ethyl 5-chloroisoxazole-3-carboxylate **23** or 5-(chloromethyl)-3-methylisoxazole **24**, K_2_CO_3_, acetonitrile, reflux; (b) dimethylamine solution 33 wt.% in H_2_O, EtOH; (c) NH_2_OH·HCl, K_2_CO_3_, EtOH_abs_, reflux; (d) (CF_3_CO)_2_O, pyridine, 85 °C.

To explore the impact of the *O*-alkyl linker modification we, firstly, exchanged an oxygen atom for a sulfur atom with conservation of the 3-carbon chain; secondly, we introduced an ester or amide group into the linker; finally, we completely replaced the alkyl linker with piperazine. Syntheses of the compounds are presented in Scheme 1, Scheme 2, Scheme 3 and Scheme 4.

In the Scheme 1, the initial benzonitrile **1** was reacted with 5-chloro-1-pentyne in the presence of potassium carbonate and potassium iodide in NMP to produce pentynylthiobenzonitrile **2**. The reaction of **2** with excess of hydroxylamine hydrochloride and potassium carbonate in absolute refluxing ethanol provided amidoxime **3** with a yield of 92%. Cyclization into 1,2,4-oxadiazole **4** was carried out using treatment of **3** with trifluoroacetic anhydride in pyridine. The cycloaddition of the S-pentyn-1 linker in **4** and 2-chloro-2-(hydroxyimino)acetic acid ethyl ester **5** or commercial acetaldoxime resulted in the target compounds **6a,b** with yields of 40% and 32%, respectively. Finally, (carbethoxy-isoxazolyl)propyl)thio)phenyl)oxadiazole **6a** reacted with dimethylamine solution in order to synthesize **7** with a yield of 57%.

2-Methyl-4-(5-(trifluoromethyl)-1,2,4-oxadiazol-3-yl)phenyl ester of 3-(3-substituted isoxazol-5-yl)propionic acids **12a,b** were synthesized according to Scheme 2. Because of the ester hydrolytic instability, synthesis of these compounds was started with formation of 1,2,4-oxadiazole cycle. Phenyloxadiazole **10** was successfully obtained via standard procedure (Scheme 1) from amidoxime **9** with a yield of 43%. The Steglich esterification of phenol **10** with 4-pentynoic acid provided **11** in 72%. In the final stage, treatment of pentynoate **11** by 2-chloro-2-(hydroxyimino)acetic acid ethyl ester **5** or acetaldoxime led to target compounds **12a,b** with low yields. Unfortunately, the reaction of **12a** with non-water dimethylamine solution did not provide the corresponding product **13**. In this case, the C-O bond of the ester breaks rapidly, forming **10**, for which the structure was confirmed by MS and NMR data.

At first, we thought of using the same sequence of steps described above (Scheme 1 and Scheme 2) for the synthesis of 2-methyl-4-(5-(trifluoromethyl)-1,2,4-oxadiazol-3-yl)phenylamide of 3-(3-substituted isoxazol-5-yl)propionic acids **20a,b** and **21**, but this was not possible for two reasons. The first reason was asphaltization of the product as a result of the amidoxime formation reaction. Yet another reason was the reaction of trifluoroacetic anhydride with the free amino group of 4-amino-*N*’-hydroxy-3-methylbenzimidamide at the second 1,2,4-oxadiazole cyclization stage. Therefore, another approach was designed.

*N*-Boc-protected aminobenzonitrile **15** was obtained from reaction of 4-amino-3-methylbenzonitrile **14** with Boc_2_O in non-solvent conditions with a yield of 93%. The consistent treatment of **15** with hydroxylamine hydrochloride and trifluoroacetic anhydride provided oxadiazole **17**. For selective cleavage of the *N*-Boc group, compound **17** was treated by trifluoroacetic acid to give aniline **18** with a yield of 99%. The reaction of **18** with 4-pentynoic acid in the presence of EDCl as dehydrating agent, DMAP as catalyst in the medium of DCM provided pentynamide **19**. Final compounds **20a,b** were synthesized as described above (Scheme 1 and Scheme 2). Unlike the ester (Scheme 2), the amide group is more hydrolytically stable. Treatment of **20a** with dimethylamine solution prepared the corresponding product **21** with a yield of 63%.

**Scheme 5:**
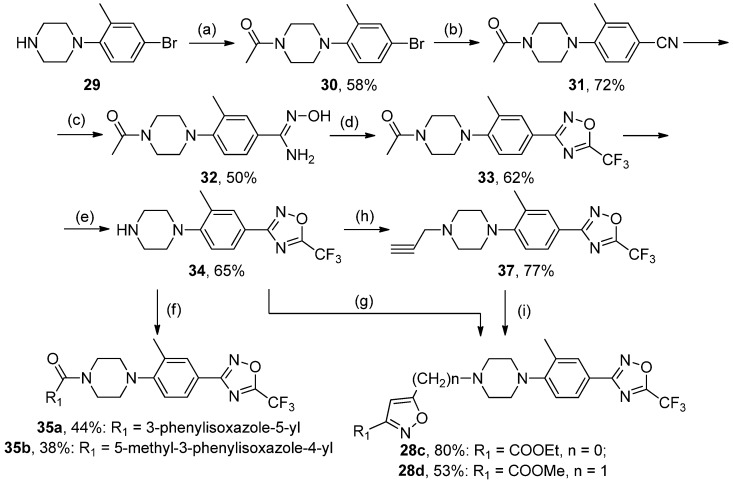
Synthesis of piperazine derivatives **35a,b**, **28c,d**. (a) Ac_2_O, 65 °C; (b) CuCN, NMP, 150 °C; (c) NH_2_OH·HCl, K_2_CO_3_, EtOH_abs_, reflux; (d) (CF_3_CO)_2_O, pyridine, 80–90 °C; (e) HCl, EtOH; (f) 3-phenylisoxazole-5-carboxylic acid or 5-methyl-3-phenylisoxazole-4-carboxylic acid, EDC, DMAP, DCM, rt; (g) 5-chloroisoxazole-3-carboxylate **23**, K_2_CO_3_, acetonitrile, reflux; (h) propargyl bromide, K_2_CO_3_, KI, acetonitrile, 50 °C; (i) 2-chloro-2-(hydroxyimino)acetic acid methyl ester **36**, Et_3_N, DMF, 80–90 °C.

Two different ways for the synthesis of piperazine derivatives were developed as indicated in Scheme 4 and Scheme 5. The initial Scheme 4 was designed based on intermediate 22, which was coupled with two isoxazoles, and in the final stages, 1,2,4-oxadiazole formation was provided. Scheme 5 was developed in order to improve Scheme 4, and in this case, the synthesis of target piperazine derivatives was dependent on the key intermediate, compound **34**, which was especially prepared for these goals.

The couplings of piperazinylbenzonitrile **22** and isoxazoles **23** or **24** in the presence of potassium carbonate in refluxing acetonitrile give the corresponding compounds **25a,b** with yields of 40% and 56%, respectively. In the case of derivative **25a**, having the carbethoxy group in the isoxazole, it was not possible to carry out the reaction of amidoxime formation directly, in our opinion, due to hydrolysis of the ester. Therefore, the ester group was replaced with the amide group using dimethylamine solution to provide **26**. In the case of **25b**, direct reaction with hydroxylamine hydrochloride successfully provided amidoxime **27b**. Finally, oxadiazole cyclization with the formation of piperazine derivatives **28a,b** took place under typical conditions (Scheme 1, Scheme 2 and Scheme 3).

In another approach, 1-(4-bromo-2-methylphenyl)piperazine **29** was *N*-protected with acetic anhydride to yield **30**. The bromine atom in **30** was exchanged to a cyano group by copper(I) cyanide in NMP at high temperature in accordance with the procedure [14]. Benzonitrile **31** was treated in two steps under typical conditions for 1,2,4-oxadiazole cyclization (Scheme 1, Scheme 2, Scheme 3 and Scheme 4) to provide compound **33** with a yield of 62%. For selective cleavage of the acetyl group, **33** was worked up with hydrochloric acid in ethanol to give ((piperazinyl)phenyl)oxadiazole **34** in 65%. While working on piperazine analogue synthesis, we came across an article in which compounds with similar structural fragments like ours (the phenyl ring is bound to piperazine, which through a carbonyl group is bound to isoxazole) were studied against influenza virus A [15]. It was interesting to investigate this structural fragment in the skeleton of our compound. For this purpose, compound **34** was coupled with commercially available 3-phenylisoxazole-5-carboxylic acid or 5-methyl-3-phenylisoxazole-4-carboxylic acid in the presence of EDCl, DMAP, and DCM to obtain the products **35a,b** with yields of 44% and 38%, respectively. Moreover, **34** was reacted with isoxazole **23** to provided **28c** in 80%. Finally, **34** was successively treated with propargyl bromide and 2-chloro-2-(hydroxyimino)acetic acid methyl ester **36** to give **28d**. The structures of the all derivatives were characterized by ^1^H-NMR spectroscopy and mass spectrometry.

The final compounds **6a,b**, **7**, **12a,b**, **20a,b**, **21**, **28a,b**, **35a,b,** and **28c,d** were tested for their activity against Coxsackievirus B3 Nancy in the viral yield reduction assay. Derivatives **35a,b** were also tested for anti-influenza A activity. Based on the results obtained, 50% cytotoxic concentration (CC_50_) and 50% inhibiting concentration (IC_50_) were calculated for each compound. The selectivity index was calculated for each compound as a ratio of CC_50_ to IC_50_. The biological results are summarized in Table 1 and Table 2 below. Pleconaril was used for comparison.

A large majority of the tested compounds (85%) were non-toxic and demonstrated CC_50_> 400 μM, except for **28c** and **28d**, which have CC_50_ values about 53.2 and 203.8 μM, respectively. Replacement of an oxygen atom with a sulfur atom in the propyl linker led to a decrease in the activity. Derivatives with the *S*-linker **6a**, **6b,** and **7**, having COOEt, Me, and CONMe_2_ groups in the isoxazole ring, respectively, demonstrated IC_50_ values of 21.0, 15.6, and 18.6 μM, while derivatives with the O-linker, having the same groups in the same position, showed IC_50_ about 18.96 [11], 4.6 [10], and 2.76 μM [11], respectively.

Insertion of the carbonyl group to the *O*-linker exhibited dissimilar results: in the case of the isoxazole COOEt group the modification increased the anti-CVB3 activity (9.1 μM for C(O)O-linker and 18.96 μM for *O*-linker), but for the methyl group, the variation greatly reduced the ability to inhibit CVB3 replication (13.1 μM for C(O)O-linker and 4.6 μM for *O*-linker).

When the carbamoyl group was introduced instead of the *O*-linker (**20a,b**), the antiviral activity was reduced again. However, for compound **21**, this change was reflected positively: this derivative has a good IC_50_ (6.8 μM) and, subsequently, the highest selectivity index (248) in this series.

Finally, we examined the impact of the propyl linker substitution to piperazine on viral inhibition. Derivatives without 1-carbon chain between piperazine and isoxazole cycles, i.e., piperazine linked to isoxazole directly, having the dimethylcarbamoyl group **28a**, are more active (SI = 205) than the same derivative with the carbethoxy group **28c** (SI = 1). On the other hand, antiviral testing of derivatives with 1-carbon chain **28b** and **28d** exhibited inconclusive results: **28b** has a poor IC_50_ and good CC_50_ values, whereas **28d** showed the highest value of IC_50_ in the series, but unfortunately **28d** was cytotoxic (CC_50_~203.8 μM). Derivative **35b** with R_1_ = 5-methyl-3-phenylisoxazole-4-yl has a selectivity index like the reference compound (SI = 125 for **35b** and 130 for pleconaril) and derivative **35a** with R_1_ = 3-phenylisoxazole-5-yl was inactive towards CVB3. Both of the compounds were inactive against the influenza virus A (see Table 2).

**Figure 2:**
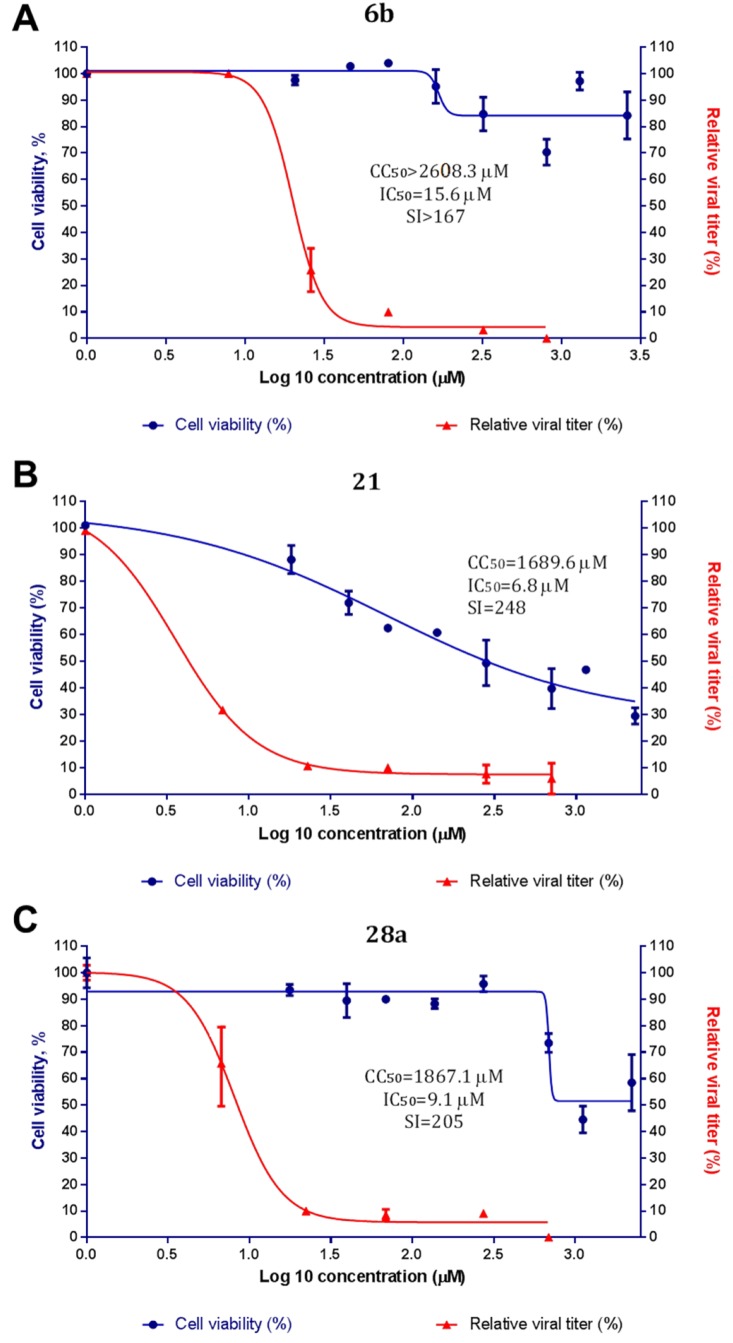
Cytotoxicity and anticoxsackievirus B3 activity of **6b** (**A**), **21** (**B**), and **28a** (**C**): logistic curves for cell viability (MTT assay) and relative viral titer (virus yield reduction assay).

The curves demonstrating the cytotoxic and virus-inhibiting properties of the most potent compounds in the series—**6b**, **21**, **28a**—are shown in Figure 2.

## 3. Materials and Methods

### 3.1. General Information

All reagents and solvents were purchased from commercial suppliers and used without further purification. ^1^H and ^13^C spectra were measured on a Bruker AC-300 (300 MHz, ^1^H) or Bruker AC-200 (50 MHz, ^13^C). Chemical shifts were measured in DMSO-d_6_ or CDCl_3_, using tetramethylsilane as an internal standard. The following abbreviations are used to indicate the multiplicity: s, singlet; d, doublet; t, triplet; q, quartet; quin, quintet; m, multiplet; dd, doublet of doublets; td, triplet of doublets; dt, doublet of triplets; ddd, doublet of doublet of doublets; bs, broad signal. Mass spectra were obtained on a Finnigan SSQ-700 with direct injection. A Waters Micromass ZQ detector was used in EI MS for identification of various products. Melting points were determined on Electrothermal 9001 (10 °C per min) and are uncorrected. Merck silica gel 60 F254 plates were used for analytical TLC; column chromatography was performed on Merck silica gel 60 (70–230 mesh).

4-mercapto-3-methylbenzonitrile **1** was obtained by the Newman–Kwart rearrangement from commercially available 4-hydroxy-3-methylbenzonitrile in three steps [16]. 4-hydroxy-3-methylbenzonitrile **8** was synthesized from corresponding phenol by *N*-bromosuccinimide (NBS) bromination and subsequent change bromine atom to cyano group by copper (I) cyanide in dry dimethylformamide [14,17]. 4-amino-3-methylbenzonitrile **14** was obtained by the method in the patent [18]. 3-methyl-4-(piperazin-1-yl)benzonitrile **22** was synthesized from 4-amino-3-methylbenzonitrile **14** and bis(2-chloroethyl)amine [19]. 1-(4-Bromo-2-methylphenyl)piperazine **29** was synthesized by the method in the article by Ge Z. et al. [20]. 2-Chloro-2-(hydroxyimino)acetic acid ethyl ester **5** or 2-chloro-2-(hydroxyimino)acetic acid methyl ester **36** were synthesized from the corresponding glycine ester hydrochloride by nitrosation with sodium nitrite and hydrochloric acid [21]. Ethyl 5-chloroisoxazole-3-carboxylate **23** was obtained by the procedure in [22]. 5-(Chloromethyl)-3-methylisoxazole **24** was synthesized according to Li W.-T. et al. [23].

### 3.2. Synthesis

#### 3.2.1. Synthesis of 3-Methyl-4-(pent-4-yn-1-ylthio)benzonitrile 2

A mixture of 4-mercapto-3-methylbenzonitrile **1** (1 mol), finely divided K_2_CO_3_ (5 mol), KI (0.01 mol), 5-chloro-1-pentyne (1.5 mol), and *N*-methylpyrrolidone-2 was heated at 65 °C for 24 h. The cooled reaction mixture was treated by cold water and stirred for 3–4 h. The solid was collected and recrystallized from methanol. Light beige solid, yield 69%, m.p. 36–38 °C. MS (EI), *m*/*z* (*I*_relat_.(%)): 215 [M]^+^ (67). Calc. 215.3140, C_13_H_13_NS. ^1^H-NMR (DMSO-*d*_6_): *δ* 1.94 (2H, quint, *J* = 7.3, CH_2_CH_2_CH_2_S), 2.25 (3H, s, CH_3_), 2.30 (2H, m, CH_2_CH_2_CH_2_S), 2.77 (1H, s, CHCCH_2_), 3.15 (2H, t, *J* = 7.3, CH_2_CH_2_CH_2_S), 7.46 (1H, d, *J* = 8.8, H6), 7.62 (1H, dd, *J* = 8.8, *J* = 0.5, H5), 7.63 (1H, s, H3) ppm.

#### 3.2.2. General Procedure for the Synthesis of Compounds **3**, **9**, **16**, **27a**,**b**, **32**

A mixture of benzonitriles **2**, **8**, **15**, **25b**, **26,** and **31** (1 mmol), finely divided K_2_CO_3_ (5 mmol), and hydroxylamine hydrochloride (5 mmol) in absolute ethanol was refluxed for 24 h. The hot reaction mixture was filtered, and the remaining solids were washed with hot acetone. The combined filtrates were concentrated in vacuo. The residue was recrystallized from the corresponding solvent (in parentheses following mp data).

*N′-Hydroxy-3-methyl-4-(pent-4-yn-1-ylthio)benzimidamide***3**, Light yellow solid, yield 92%, m.p. 64–66 °C (EtOH). MS (EI), *m*/*z* (*I_relat._*(%)): 248 [M]^+^ (54). Calc. 248.3439, C_13_H_16_N_2_OS. ^1^H-NMR (DMSO-*d*_6_): *δ* 1.86 (2H, quint, *J* = 7.3, CH_2_CH_2_CH_2_S), 2.37 (2H, t, *J* = 7.3, CH_2_CH_2_CH_2_S), 2.42 (3H, s, CH_3_Ph), 2.77 (1H, s, CHCCH_2_), 3.25 (2H, t, *J* = 7.3, CH_2_CH_2_CH_2_S), 4.96 (1H, s, NOH), 5.05 (2H, brs, NH_2_), 7.13 (1H, d, *J* = 8.8, H6), 7.29 (1H, dd, *J* = 8.8, *J* = 0.5, H5), 7.33 (1H, s, H3) ppm.

*N′,4-dihydroxy-3-methylbenzimidamide***9**, Light beige solid, yield 35%, m.p. 70–71 °C (EtOH). MS (EI), *m*/*z* (*I_relat_.*(%)): 166 [M]^+^ (100). Calc. 166.1772, C_8_H_10_N_2_O_2_. ^1^H-NMR (DMSO-*d*_6_): *δ* 2.30 (3H, s, CH_3_Ph), 4.99 (1H, s, NOH), 5.09 (2H, brs, NH_2_), 6.83 (1H, d, *J* = 7.5, H6), 7.34 (1H, d, *J* = 7.5, H5), 7.51 (1H, s, H3) ppm.

*Tert-butyl (4-(N′-hydroxycarbamimidoyl)-2-methylphenyl)carbamate***16,** White solid, yield 58%, m.p. 74–76 °C (decomp.) (iPrOH). MS (EI), *m*/*z* (*I_relat_.*(%)): 265 [M]^+^ (83). Calc. 265.3083, C_13_H_19_N_3_O_3_. ^1^H-NMR (DMSO-*d_6_*): *δ* 1.43 (9H, s, tBu), 2.22 (3H, s, CH_3_Ph), 4.94 (1H, s, NOH), 5.03 (2H, brs, NH_2_), 6.81 (1H, d, *J* = 7.5, H6), 7.24 (1H, d, *J* = 7.5, H5), 7.33 (1H, s, H3) ppm.

*5-(4-(4-(N′-Hydroxycarbamimidoyl)-2-methylphenyl)piperazin-1-yl)-N,N-dimethylisoxazole-3-carboxamide***27a**, White solid, yield 62%, mp 203–205 °C (EtOH). MS (EI), *m*/*z* (*I_relat._*(%)): 372 [M]^+^ (67). Calc. 372.4216, C_18_H_24_N_6_O_3_. ^1^H-NMR (DMSO-*d*_6_): *δ* 2.22 (3H, s, CH_3_Ph), 2.73 (6H, s, N(CH_3_)_2_), 3.24 (4H, brt, N(CH_2_)_2_), 3.21 (4H, brt, N(CH_2_)_2_), 4.94 (1H, s, NOH), 5.01 (2H, brs, NH_2_), 5.80 (1H, s, isoxazole), 6.46 (1H, d, *J* = 8.0, H6), 7.26 (1H, d, *J* = 8.0, H5), 7.27 (1H, s, H3) ppm.

*N′-hydroxy-3-methyl-4-(4-((3-methylisoxazol-5-yl)methyl)piperazin-1-yl)benzimidamide***27b**, White solid, yield 47%, m.p. 169–170 °C (EtOH). MS (EI), *m*/*z* (*I_relat._*(%)): 329 [M]^+^ (54). Calc. 329.3968, C_17_H_23_N_5_O_2_. ^1^H-NMR (DMSO-*d*_6_): *δ* 2.22 (3H, s, CH_3_), 2.30 (3H, s, CH_3_Ph), 2.73 (4H, m, N(CH_2_)_2_), 3.07 (4H, brt, N(CH_2_)_2_), 4.12 (2H, brs, NCH_2_), 4.96 (1H, s, NOH), 5.01 (2H, brs, NH_2_), 6.30 (1H, s, isoxazole), 6.46 (1H, d, *J* = 9.0, H6), 7.26 (1H, d, *J* = 9.0, H5), 7.27 (1H, s, H3) ppm.

*4-(4-Acetylpiperazin-1-yl)-N′-hydroxy-3-methylbenzimidamide***32**, White solid, yield 50%, m.p. 230–232 °C (MeOH). MS (EI), *m*/*z* (*I_relat._*(%)): 276 [M]^+^ (61). Calc. 276.3342, C_14_H_20_N_4_O_2_. ^1^H-NMR (DMSO-*d*_6_): *δ* 1.93 (3H, s, CH_3_), 2.22 (3H, s, CH_3_Ph), 3.29 (4H, brs, N(CH_2_)_2_), 3.63 (4H, brs, N(CH_2_)_2_), 4.96 (1H, s, NOH), 5.03 (2H, brs, NH_2_), 6.46 (1H, d, *J* = 7.9, H6), 7.26 (1H, d, *J* = 7.9, H5), 7.27 (1H, s, H3) ppm.

#### 3.2.3. General Procedure for the Synthesis of Compounds **4**, **10**, **17**, **28a**,**b**, **33**

To a solution of **3**, **9**, **16**, **27a,b,** or **32** (1 mmol) in of pyridine heated to 80–90 °C carefully add dropwise trifluoroacetic anhydride (2 mmol) during 30 min. The reaction mixture was stored for 1 h at 85 °C. The cooled to rt mixture was diluted with water and extracted with ethyl acetate (3 times). The combined organic phases were washed with water (3 times), dried over anhydrous Na_2_SO_4_, and concentrated in vacuo. The residue was treated by water and stored in the refrigerator for 2–4 h. Crystals were collected and recrystallized from the corresponding solvent (in parentheses following mp data).

*3-(3-Methyl-4-(pent-4-yn-1-ylthio)phenyl)-5-(trifluoromethyl)-1,2,4-oxadiazole***4**, White solid, yield 54%, mp 49–50 °C (iPrOH). MS (EI), *m*/*z* (*I_relat._*(%)): 326 [M]^+^ (76). Calc. 326.3367, C_15_H_13_F_3_N_2_OS. ^1^H-NMR (DMSO-*d*_6_): *δ* 1.86 (2H, quint, *J* = 7.2, CH_2_CH_2_CH_2_S), 2.21 (3H, s, CH_3_Ph), 2.37 (2H, t, *J* = 7.2, CH_2_CH_2_CH_2_S), 2.77 (1H, s, CHCCH_2_), 3.25 (2H, t, *J* = 7.2, CH_2_CH_2_CH_2_S), 7.47 (1H, d, *J* = 7.5, H6), 7.59 (1H, d, *J* = 7.5, H5), 7.64 (1H, s, H3) ppm.

*2-Methyl-4-(5-(trifluoromethyl)-1,2,4-oxadiazol-3-yl)phenol***10**, White solid, yield 43%, m.p. 64–65 °C (Hexane). MS (EI), *m*/*z* (*I_relat._*(%)): 244 [M]^+^ (64). Calc. 244.1700, C_10_H_7_F_3_N_2_O_2_. ^1^H-NMR (DMSO-*d*_6_): *δ* 2.19 (3H, s, CH_3_Ph), 7.05 (1H, d, *J* = 7.5, H6), 7.62 (1H, d, *J* = 7.5, H5), 7.99 (1H, s, H3) ppm.

*Tert-butyl (2-methyl-4-(5-(trifluoromethyl)-1,2,4-oxadiazol-3-yl)phenyl)carbamate***17**, White solid, yield 48%, m.p. 104–106 °C (iPrOH). MS (EI), *m*/*z* (*I_relat._*(%)): 343 [M]^+^ (58). Calc. 343.3010, C_15_H_16_F_3_N_3_O_3_. ^1^H-NMR (DMSO-*d*_6_): *δ* 1.43 (9H, s, tBu), 2.16 (3H, s, CH_3_Ph), 7.53 (1H, d, *J* = 7.5, H6), 7.62 (1H, d, *J* = 7.5, H5), 7.65 (1H, s, H3) ppm.

*N,N-Dimethyl-5-(4-(2-methyl-4-(5-(trifluoromethyl)-1,2,4-oxadiazol-3-yl)phenyl)piperazin-1-yl)iso-xazole-3-carboxamide***28a**, White solid, yield 59%, m.p. 171–173 °C (EtOH). MS (EI), *m*/*z* (I_relat._(%)): 450 [M]^+^ (53). Calc. 450.4143, C_20_H_21_F_3_N_6_O_3_. ^1^H NMR (DMSO-d_6_): δ 2.39 (3H, s, CH_3_Ph), 2.98 (3H, s, NCH_3_), 3.04–3.11 (7H, m, N(CH_2_)_2_, NCH_3_), 3.52 (4H, brt, N(CH_2_)_2_), 5.54 (1H, s, isoxazole), 7.23 (1H, d, *J* = 8.0, H6), 7.86 (1H, d, *J* = 8.0, H5), 7.87 (1H, s, H3) ppm. ^13^C-NMR (DMSO-d_6_): δ 17.88, 35.15, 35.15, 46.54, 46.54, 49.96, 49.96, 86.16, 115.89 (q, *J* = 273.4), 118.10, 120.00, 126.97, 128.15, 129.03, 149.89, 156.44, 163.02, 166.00, 167.12 (q, *J* = 43.0), 171.16 ppm.

*3-(3-Methyl-4-(4-((3-methylisoxazol-5-yl)methyl)piperazin-1-yl)phenyl)-5-(trifluoromethyl)-1,2,4-oxadiazole***28b**, White solid, yield 31%, mp 94–96 °C (CCl_4_). MS (EI), *m*/*z* (*I_relat._*(%)): 407 [M]^+^ (48). Calc. 407.3896, C_19_H_20_F_3_N_5_O_2_. ^1^H-NMR (DMSO-*d*_6_): *δ* 2.28 (3H, s, CH_3_), 2.34 (3H, s, CH_3_Ph), 3.14 (4H, m, N(CH_2_)_2_), 3.52 (4H, brt, N(CH_2_)_2_), 4.37 (2H, brs, NCH_2_), 6.55 (1H, s, isoxazole), 7.21 (1H, d, *J* = 9.0, H6), 7.86 (1H, d, *J* = 9.0, H5), 7.87 (1H, s, H3) ppm. ^13^C-NMR (DMSO-*d*_6_): *δ* 11.26, 17.81, 50.00, 50.00, 52.11, 52.79, 52.79, 103.88, 115.23 (q, *J* = 273.1), 118.17, 120.00, 127.61, 128.68, 129.80, 149.91, 159.00, 165.99, 166.00, 167.15 (q, *J* = 43.5) ppm.

*1-(4-(2-methyl-4-(5-(trifluoromethyl)-1,2,4-oxadiazol-3-yl)phenyl)piperazin-1-yl)ethanone***33**, White solid, yield 62%, m.p. 65–67 °C (Hexane). MS (EI), *m*/*z* (*I_relat._*(%)): 354 [M]^+^ (59). Calc. 354.3270, C_16_H_17_F_3_N_4_O_2_. ^1^H-NMR (DMSO-*d*_6_): *δ* 1.93 (3H, s, CH_3_), 2.21 (3H, s, CH_3_Ph), 3.30 (4H, brs, N(CH_2_)_2_), 3.63 (4H, brs, N(CH_2_)_2_), 7.00 (1H, d, *J* = 7.9, H6), 7.43 (1H, d, *J* = 7.9, H5), 7.56 (1H, s, H3) ppm.

#### 3.2.4. General Procedure for the Synthesis of Compounds **6a**, **12a**, **20a**, **28d**

To a solution of **5** or **36** (3 mmol) in DMF, a solution of corresponding compounds **4**, or **11**, or **19**, or **37** in DMF for 20–30 min was added and stirred at rt for 40–50 min. To the reaction solution, Et_3_N (3 mmol) in DMF was added at 80–90 °C for 2 h. The mixture was stirred at 80–90 °C for 1–2 h and at rt for 12 h. The reaction mixture was diluted with water and extracted with ethyl acetate (3-times). The combined organic phases were washed with water (3-times), dried over anhydrous Na_2_SO_4_, and concentrated in vacuo. The residue was treated by water and stored in the refrigerator for 2–4 h. Crystals were collected and recrystallized from the corresponding solvent (in parentheses following mp data).

*3-(3-Methyl-4-((3-(3-carbethoxy-isoxazol-5-yl)propyl)thio)phenyl)-5-(trifluoromethyl)-1,2,4-oxadia-zole***6a**, White solid, yield 40%, m.p. 97–100 °C (MeOH). MS (EI), *m*/*z* (*I_relat._*(%)): 441 [M]^+^ (65). Calc. 441.4241, C_19_H_18_F_3_N_3_O_4_S. ^1^H-NMR (DMSO-*d*_6_): *δ* 1.27 (3H, t, *J* = 7.1, CH_3_CH_2_O), 2.05 (2H, quint, *J* = 7.5, *J* = 7.4, CH_2_CH_2_CH_2_S), 2.31 (3H, s, CH_3_Ph), 3.01 (2H, t, *J* = 7.5, CH_2_CH_2_CH_2_S), 3.11 (2H, t, *J* = 7.5, CH_2_CH_2_CH_2_S), 4.32 (2H, q, *J* = 7.1, CH_3_CH_2_O), 6.74 (1H, s, isoxazole), 7.47 (1H, d, *J* = 8.5, H6), 7.84 (2H, d, *J* = 8.5, H3, H5) ppm. ^13^C-NMR (DMSO-*d*_6_): *δ* 13.87, 19.49, 25.03, 25.97, 29.79, 61.67, 101.95, 115.76 (q, *J* = 273.7), 120.39, 125.38, 125.61, 128.15, 136.27, 141.90, 156.09, 159.50, 164.60 (q, *J* = 43.0), 168.24, 174.67 ppm.

*Ethyl 5-(3-(2-methyl-4-(5-(trifluoromethyl)-1,2,4-oxadiazol-3-yl)phenoxy)-3-oxopropyl)-isoxazole-3-carboxylate***12a**, White solid, yield 34%, m.p. 95–96 °C (Hexane). MS (EI), *m*/*z* (*I_relat._*(%)): 439 [M]^+^ (41). Calc. 439.3420, C_19_H_16_F_3_N_3_O_6_. ^1^H-NMR (DMSO-*d*_6_): *δ* 1.32 (3H, t, *J* = 7.1, CH_3_CH_2_O), 2.20 (3H, s, CH_3_Ph), 3.20–3.27 (4H, m, CH_2_CH_2_CO), 4.37 (2H, q, *J* = 7.1, CH_3_CH_2_O), 6.79 (1H, s, isoxazole), 7.33 (1H, d, *J* = 8.4, H6), 7.94 (1H, d, *J* = 8.4, H5), 8.01 (1H, s, H3) ppm. ^13^C-NMR (DMSO-*d*_6_): *δ* 13.64, 15.52, 21.43, 31.14, 60.95, 102.05, 115.84 (q, *J* = 273.6), 121.94, 122.20, 123.45, 126.23, 130.13, 131.78, 152.16, 159.88, 166.10 (q, *J* = 42.8), 167.84, 169.66, 171.15 ppm.

*Ethyl 5-(3-((2-methyl-4-(5-(trifluoromethyl)-1,2,4-oxadiazol-3-yl)phenyl)amino)-3-oxopropyl)isoxa-zole-3-carboxylate***20a**, White solid, yield 78%, m.p. 166–168 °C (EtOH). MS (EI), *m*/*z* (*I_relat._*(%)): 438 [M]^+^ (56). Calc. 438.3573, C_19_H_17_F_3_N_4_O_5_. ^1^H-NMR (DMSO-*d*_6_):*δ* 1.31 (3H, t, *J* = 7.1, CH_3_CH_2_O), 2.31 (3H, s, CH_3_Ph), 2.89 (2H, t, *J* = 7.1, CH_2_CH_2_CO), 3.17 (2H, t, *J* = 7.1, CH_2_CH_2_CO), 4.36 (2H, q, *J* = 7.1, CH_3_CH_2_O), 6.70 (1H, s, isoxazole), 7.79 (1H, d, *J* = 8.8, H6), 7.87 (1H, dd, *J* = 1.8, 8.8, H5), 7.91 (1H, s, H3), 9.53 (1H, brs, NH) ppm. ^13^C-NMR (DMSO-*d*_6_): *δ* 13.88, 17.69, 21.97, 32.91, 61.70, 101.76, 115.73 (q, *J* = 273.4), 120.31, 124.54, 125.31, 129.29, 131.62, 140.21, 156.05, 159.50, 164.84 (q, *J* = 43.9), 168.14, 169.66, 174.86 ppm.

*Methyl 5-(4-(2-methyl-4-(5-(trifluoromethyl)-1,2,4-oxadiazol-3-yl)phenyl)piperazin-1-yl)-isoxazole--3-carboxylate***28d**, White solid, yield 53%, m.p. 113–115 °C (EtOH). MS (EI), *m*/*z* (*I_relat._*(%)): 451 [M]^+^ (72). Calc. 451.3991, C_20_H_20_F_3_N_5_O_4_. ^1^H-NMR (DMSO-*d*_6_): *δ* 2.31 (3H, s, CH_3_Ph), 2.64 (4H, brs, N(CH_2_)_2_), 2.97 (4H, brs, N(CH_2_)_2_), 3.87 (2H, s, PhCH_2_N), 3.90 (3H, s, CH_3_O), 6.87 (1H, s, isoxazole), 7.17 (1H, d, *J* = 8.4, H6), 7.83 (1H, d, *J* = 8.4, H5), 7.84 (1H, s, H3) ppm. ^13^C-NMR (DMSO-*d*_6_): *δ* 17.89, 49.87, 49.87, 52.15, 52.81, 52.81, 53.11, 104.05, 115.34 (q, *J* = 273.2), 118.14, 120.03, 127.67, 128.57, 129.85, 150.23, 159.12, 160.31, 166.05, 166.14, 167.32 (q, *J* = 43.3) ppm.

#### 3.2.5. General Procedure for the Synthesis of Compounds **6b**, **12b**, **20b**

To a solution of NCS (2.5 mmol) and 1–2 drops of pyridine in DMF, a solution of acetaldoxime (2.5 mmol) in DMF was added for 30 min and stirred at rt for 1 h; then, to the solution, a solution of **4**, or **11**, or **19** (1 mmol) in DMF was added for 20 min. To the resulted mixture, Et_3_N (2.5 mmol) in DMF was added at 80–90 °C for 1 h and stirred at 80–90 °C for 3–4 h. The reaction mixture was diluted with water and extracted with ethyl acetate (3 times). The combined organic phases were washed with water (3 times), dried over anhydrous Na_2_SO_4_, and concentrated in vacuo. The residue was recrystallized from the corresponding solvent (in parentheses following mp data).

*3-(3-Methyl-4-((3-(3-methylisoxazol-5-yl)propyl)thio)phenyl)-5-(trifluoromethyl)-1,2,4-oxadiazole***6b**, White solid, yield 32%, m.p. 60–63 °C (Hexane). MS (EI), *m*/*z* (*I_relat._*(%)): 383 [M]^+^ (41). Calc. 383.3880, C_17_H_16_F_3_N_3_O_2_S. ^1^H-NMR (DMSO-*d*_6_): *δ* 2.00 (2H, quint, *J* = 7.2, CH_2_CH_2_CH_2_S), 2.18 (3H, s, CH_3_), 2.35 (3H, s, CH_3_Ph), 2.89 (2H, t, *J* = 7.2, CH_2_CH_2_CH_2_S), 3.13 (2H, t, *J* = 7.2, CH_2_CH_2_CH_2_S), 6.14 (1H, s, isoxazole), 7.48 (1H, d, *J* = 7.5, H6), 7.85 (1H, d, *J* = 7.5, H5), 7.87 (1H, s, H3) ppm. ^13^C-NMR (DMSO-*d*_6_): *δ* 11.05, 19.53, 24.96, 26.04, 29.84, 101.76, 115.78 (q, *J* = 273.1), 120.38, 125.40, 125.62, 128.19, 136.25, 141.92, 159.95, 164.71 (q, *J* = 43.4), 168.25, 170.65 ppm.

*2-Methyl-4-(5-(trifluoromethyl)-1,2,4-oxadiazol-3-yl)phenyl 3-(3-methylisoxazol-5-yl)propanoate***12b**, White solid, yield 29%, mp 67–69 °C (Hexane). MS (EI), **m*/*z** (*I_relat._*(%)): 381 [M]^+^ (53). Calc. 381.3060, C_17_H_14_F_3_N_3_O_4_. ^1^H-NMR (DMSO-*d*_6_):*δ* 2.18 (3H, s, CH_3_), 2.35 (3H, s, CH_3_Ph), 3.20–3.27 (4H, m, CH_2_CH_2_CO), 6.14 (1H, s, isoxazole), 7.48 (1H, d, *J* = 7.5, H6), 7.85 (1H, d, *J* = 7.5, H5), 7.87 (1H, s, H3) ppm. ^13^C-NMR (DMSO-*d*_6_): *δ* 10.89, 15.48, 21.45, 31.06, 102.16, 115.77 (q, *J* = 273.4), 122.15, 123.44, 126.34, 130.05, 131.67, 152.12, 159.46, 165.01 (q, *J* = 42.8), 167.92, 169.86, 170.97 ppm.

*N-(2-Methyl-4-(5-(trifluoromethyl)-1,2,4-oxadiazol-3-yl)phenyl)-3-(3-methylisoxazol-5-yl)propan-amide***20b**, White solid, yield 83%, m.p. 176–179 °C (EtOH). MS (EI), *m*/*z* (*I_relat._*(%)): 380 [M]^+^ (68). Calc. 380.3212, C_17_H_15_F_3_N_4_O_3_. ^1^H-NMR (DMSO-*d*_6_): *δ* 2.19 (3H, s, CH_3_), 2.31 (3H, s, CH_3_Ph), 2.82 (2H, t, *J* = 7.3, CH_2_CH_2_CO), 3.05 (2H, t, *J* = 7.3, CH_2_CH_2_CO), 6.13 (1H, s, isoxazole), 7.81 (1H, d, *J* = 8.5, H6), 7.87 (1H, dd, *J* = 1.8, 8.5, H5), 7.91 (1H, s, H3), 9.49 (1H, brs, NH) ppm. ^13^C-NMR (DMSO-*d*_6_): *δ* 11.15, 17.68, 21.87, 33.12, 100.97, 115.75 (q, *J* = 273.1), 120.33, 124.45, 125.30, 129.27, 121.67, 140.15, 159.86, 164.80 (q, *J* = 43.5), 168.10, 169.69, 173.97 ppm.

#### 3.2.6. General Procedure for the Synthesis of Compounds **7**, **21**, **26**

A mixture of **6a**, or **20a**, or **25a** and dimethylamine solution 17 wt.% in dioxane was heated at 50–60 °C for 1–12 h. The cooled reaction mixture was concentrated in vacuo. The residue was treated by water and stored in the refrigerator for 12 h. Crystals were collected and recrystallized from the corresponding solvent (in parentheses following mp data).

*N,N-dimethyl-5-(3-((2-methyl-4-(5-(trifluoromethyl)-1,2,4-oxadiazol-3-yl)phenyl)thio)propyl)isoxa-zole-3-carboxamide***7**, White solid, yield 57%, m.p. 95.5–97 °C (Hexane). MS (EI), *m*/*z* (*I_relat._*(%)): 440 [M]^+^ (84). Calc. 440.4394, C_19_H_19_F_3_N_4_O_3_S. ^1^H-NMR (DMSO-*d*_6_): *δ*2.05 (2H, quint, *J* = 7.2, CH_2_CH_2_CH_2_S), 2.34 (3H, s, CH_3_Ph), 2.99 (3H, s, NCH_3_), 3.05 (3H, s, NCH_3_), 3.02 (2H, t, *J* = 7.2, CH_2_CH_2_CH_2_S), 3.15 (2H, t, *J* = 7.2, CH_2_CH_2_CH_2_S), 6.52 (1H, s, isoxazole), 7.47 (1H, s, *J* = 7.5, H6), 7.85 (1H, d, *J* = 7.5, H5), 7.87 (1H, s, H3) ppm. ^13^C-NMR (DMSO-*d*_6_): *δ* 19.50, 24.94, 26.07, 29.86, 34.85, 37.95, 101.66, 115.77 (q, *J* = 273.3), 120.40, 125.40, 125.59, 128.17, 136.28, 141.94, 158.70, 160.58, 164.85 (q, *J* = 43.5), 168.24, 172.74 ppm.

*N,N-dimethyl-5-(3-((2-methyl-4-(5-(trifluoromethyl)-1,2,4-oxadiazol-3-yl)phenyl)amino)-3-oxopro-pyl)isoxazole-3-carboxamide***21**, White solid, yield 63%, m.p. 169–171 °C (Hexane:EtOAc). MS (EI), *m*/*z* (I_relat._(%)): 437 [M]^+^ (63). Calc. 437.3725, C_19_H_18_F_3_N_5_O_4_. ^1^H-NMR (DMSO-d_6_): δ 2.31 (3H, s, CH_3_Ph), 2.88 (2H, t, *J* = 7.2, CH_2_CH_2_CO), 3.00 (3H, s, NCH_3_), 3.11 (3H, s, NCH_3_), 3.15 (2H, t, *J* = 7.2, CH_2_CH_2_CO), 6.49 (1H, s, isoxazole), 7.80 (1H, d, *J* = 8.3, H6), 7.87 (1H, dd, *J* = 2.0, 8.3, H5), 7.91 (1H, s, H3), 9.53 (1H, brs, NH) ppm. ^13^C-NMR (DMSO-d_6_): δ 17.66, 21.82, 32.99, 34.82, 37.86, 101.48, 115.72 (q, *J* = 273.2), 120.25, 124.49, 125.25, 129.22, 131.61, 140.17, 158.61, 160.48, 164.79 (q, *J* = 43.0), 168.09, 169.64, 172.79 ppm.

*5-(4-(4-Cyano-2-methylphenyl)piperazin-1-yl)-N,N-dimethylisoxazole-3-carboxamide***26**, White solid, yield 77%, m.p. 145–147 °C (EtOH). MS (EI), *m*/*z* (*I_relat_.*(%)): 339 [M]^+^ (72). Calc. 339.3916, C_18_H_21_N_5_O_2_. ^1^H-NMR (DMSO-*d*_6_): *δ* 2.29 (3H, s, CH_3_Ph), 2.73 (6H, s, N(CH_3_)_2_), 3.20 (4H, brt, N(CH_2_)_2_), 3.21 (4H, brt, N(CH_2_)_2_), 5.80 (1H, s, isoxazole), 6.60 (1H, d, *J* = 8.0, H6), 7.49 (1H, d, *J* = 8.0, H5), 7.64 (1H, s, H3) ppm.

#### 3.2.7. Synthesis of 2-Methyl-4-(5-(trifluoromethyl)-1,2,4-oxadiazol-3-yl)phenyl pent-4-ynoate 11

A mixture of **10** (1 mmol), DCC (2 mmol), 4-pentynoic acid (2 mmol) in pyridine was stirred at rt for 12 h. The mixture was diluted with CHCl_3_, and precipitated urea was filtered off. The CHCl_3_ solution was washed with 3% aq. HCl and water (3 times) and then dried over Na_2_SO_4_. The solution was filtered off through a short silica gel column, and the solvent was concentrated in vacuo. White solid, yield 72%, m.p. 54–56 °C. MS (EI), *m*/*z* (*I_relat._*(%)): 324 [M]^+^ (78). Calc. 324.2546, C_15_H_11_F_3_N_2_O_3_. ^1^H-NMR (DMSO-*d*_6_): *δ* 2.22 (3H, s, CH_3_Ph), 2.51–2.56 (2H, quint, CH_2_CH_2_CO), 2.82 (2H, m, CH_2_CH_2_O), 2.86 (1H, s, CHCCH_2_), 7.32 (1H, d, *J* = 8.4, H6), 7.94 (1H, d, *J* = 8.4, H5), 8.01 (1H, s, H3) ppm.

#### 3.2.8. Synthesis of *tert*-Butyl (4-cyano-2-methylphenyl)carbamate 15

A mixture of **14** (1 mol) and Boc_2_O (3 mol) was refluxed for 48 h. The reaction mixture was diluted with methanol, brought to the boil, and concentrated in vacuo. The procedure was repeated 3 times. The residue was treated by hexane and stored in the refrigerator for 4–6 h. Crystals were collected and recrystallized from ethanol. White solid, yield 93%, mp 89–91 °C. MS (EI), *m*/*z* (*I_relat._*(%)): 232 [M]^+^ (37). Calc. 232.2783, C_13_H_16_N_2_O_2_. ^1^H-NMR (DMSO-*d*_6_): *δ* 1.43 (9H, s, tBu), 2.28 (3H, s, CH_3_Ph), 6.88 (1H, d, *J* = 7.5, H6), 7.60 (1H, d, *J* = 7.5, H5), 7.74 (1H, s, H3) ppm.

#### 3.2.9. Synthesis of 2-Methyl-4-(5-(trifluoromethyl)-1,2,4-oxadiazol-3-yl)aniline 18

To a solution of **17** (1 mmol) in DCM, TFA (10 mmol) was added at 0 °C. The reaction mixture was stirred at rt for 2–3 h, after which the solvents were removed in vacuo. The residue was triturated with water to provide the product as a solid. Crystals were collected and recrystallized from hexane:EtOAc. White solid, yield 99%, m.p. 118 °C (decomp.). MS (EI), *m*/*z* (*I_relat._*(%)): 243 [M]^+^ (81). Calc. 243.1852, C_10_H_8_F_3_N_3_O. ^1^H-NMR (DMSO-*d*_6_): *δ* 2.23 (3H, s, CH_3_Ph), 5.27 (2H, brs, NH_2_), 6.71 (1H, d, *J* = 7.5, H6), 7.58 (1H, d, *J* = 7.5, H5), 7.64 (1H, s, H3) ppm.

#### 3.2.10. Synthesis of *N*-(2-Methyl-4-(5-(trifluoromethyl)-1,2,4-oxadiazol-3-yl)phenyl)pent-4-ynamide 19

To a solution of **18** (1 mol) and DMAP (1.3 mol) in DCM, 4-pentynoic acid (1 mol) was added in one portion, followed by the addition of EDCI (1.3 mol) in one portion at rt. The reaction mixture was stirred at rt overnight, after which it was washed successively with 3% aq. HCl and water (3 times). The organic layer was dried over Na_2_SO_4_ and concentrated in vacuo. The residue was recrystallized from ethanol. White solid, yield 38%, m.p. 165–167 °C. MS (EI), *m*/*z* (*I_relat._*(%)): 323 [M]^+^ (65). Calc. 323.2699, C_15_H_12_F_3_N_3_O_2_. ^1^H-NMR (DMSO-*d*_6_): *δ* 2.16 (3H, s, CH_3_Ph), 2.50–2.57 (2H, quint, CH_2_CH_2_CO), 2.62 (2H, m, CH_2_CH_2_O), 2.78 (1H, s, CHCCH_2_), 7.32 (1H, d, *J* = 8.4, H6), 7.61 (1H, d, *J* = 8.4, H5), 7.70 (1H, s, H3), 9.12 (1H, brs, NH) ppm.

#### 3.2.11. General Procedure for the Synthesis of Compounds **25a**,**b**, **28c**

A mixture of the corresponding phenyl-piperazine (1 mol), the corresponding isoxazole (1.4 mol), and finely divided K_2_CO_3_ (3 mol) in acetonitrile was refluxed for 24–48 h. The hot mixture was filtered, and the remaining solids were washed with acetonitrile. The combined filtrates were concentrated in vacuo. The residue was recrystallized from ethanol.

*Ethyl 5-(4-(4-cyano-2-methylphenyl)piperazin-1-yl)isoxazole-3-carboxylate***25a**, Light beige solid, yield 40%, m.p. 124–125 °C. MS (EI), *m*/*z* (*I_relat._*(%)): 340 [M]^+^ (42). Calc. 340.3764, C_18_H_20_N_4_O_3_. ^1^H-NMR (DMSO-*d*_6_): *δ* 1.29 (3H, t, *J* = 7.1, CH_3_CH_2_O), 2.32 (3H, s, CH_3_Ph), 3.05 (4H, brs, N(CH_2_)_2_), 3.52 (4H, brs, N(CH_2_)_2_), 4.35 (2H, q, *J* = 7.1, CH_3_CH_2_O), 5.75 (1H, s, isoxazole), 7.19 (1H, d, *J* = 7.9, H6), 7.61 (1H, d, *J* = 7.9, H5), 7.63 (1H, s, H3) ppm.

*3-Methyl-4-(4-((3-methylisoxazol-5-yl)methyl)piperazin-1-yl)benzonitrile***25b**, Yellow solid, yield 56%, m.p. 86–88 °C. MS (EI), *m*/*z* (*I_relat._*(%)): 296 [M]^+^ (57). Calc. 296.3669, C_17_H_20_N_4_O. ^1^H-NMR (DMSO-*d*_6_): *δ* 2.29 (3H, s, CH_3_), 2.30 (3H, s, CH_3_Ph), 2.72 (4H, m, N(CH_2_)_2_), 3.02 (4H, m, N(CH_2_)_2_), 4.12 (2H, brs, NCH_2_), 6.30 (1H, s, isoxazole), 6.60 (1H, d, *J* = 9.0, H6), 7.49 (1H, d, *J* = 9.0, H5), 7.64 (1H, s, H3) ppm.

*Ethyl 5-(4-(2-methyl-4-(5-(trifluoromethyl)-1,2,4-oxadiazol-3-yl)phenyl)piperazin-1-yl)isoxazole-3-carboxylate***28c**, White solid, yield 80%, m.p. 121–123 °C. MS (EI), *m*/*z* (*I_relat._*(%)): 451 [M]^+^ (46). Calc. 451.3991, C_20_H_20_F_3_N_5_O_4_. ^1^H-NMR (DMSO-*d*_6_):*δ* 1.30 (3H, t, *J* = 7.1, CH_3_CH_2_O), 2.38 (3H, s, CH_3_Ph), 3.07 (4H, brs, N(CH_2_)_2_), 3.54 (4H, brs, N(CH_2_)_2_), 4.32 (2H, q, *J* = 7.1, CH_3_CH_2_O), 5.80 (1H, s, isoxazole), 7.23 (1H, d, *J* = 7.9, H6), 7.86 (1H, d, *J* = 7.9, H5), 7.87 (1H, s, H3) ppm. ^13^C-NMR (DMSO-*d*_6_): *δ* 13.89, 17.82, 46.58, 46.58, 49.94, 49.94, 61.75, 87.21, 115.73 (q, *J* = 273.2), 118.15, 120.05, 126.94, 128.19, 128.95, 150.07, 156.42, 160.34, 162.95, 167.10 (q, *J* = 43.1), 171.05 ppm.

#### 3.2.12. Synthesis of 1-(4-(4-Bromo-2-methylphenyl)piperazin-1-yl)ethanone 30

A mixture of **19** (1 mol) and Ac_2_O (5 mol) was heated at 60–65 °C for 4–5 h. The reaction mixture was poured into cold water and stirred for 2 h. Precipitate was collected and recrystallized from methanol. White solid, yield 58%, m.p. 120–121 °C. MS (EI), *m*/*z* (*I_relat._*(%)): 297 [M]^+^ (73). Calc. 297.1909, C_13_H_17_BrN_2_O. ^1^H-NMR (DMSO-*d*_6_): *δ* 1.93 (3H, s, CH_3_), 2.14 (3H, s, CH_3_Ph), 3.12 (4H, brs, N(CH_2_)_2_), 3.63 (4H, brs, N(CH_2_)_2_), 6.47 (1H, d, *J* = 7.9, H6), 6.83 (1H, d, *J* = 7.9, H5), 6.85 (1H, s, H3) ppm.

#### 3.2.13. Synthesis of 4-(4-Acetylpiperazin-1-yl)-3-methylbenzonitrile 31

A mixture of **30** (1 mol) and CuCN (1.4. mol) in NMP was heated at 150 °C for 4 h. The cool mixture was poured into 3% aq. HCl and diluted with ethyl acetate (3 times). The combined organic layers were washed with water (3 times), dried over Na_2_SO_4_, and concentrated in vacuo. The residue was triturated with water and collected. White solid, yield 72%, m.p. 230–232 °C. MS (EI), *m*/*z* (*I_relat._*(%)): 243 [M]^+^ (69). Calc. 243.3043, C_14_H_17_N_3_O. ^1^H NMR (DMSO-*d*_6_): *δ* 1.93 (3H, s, CH_3_), 2.29 (3H, s, CH_3_Ph), 3.32 (4H, brs, N(CH_2_)_2_), 3.63 (4H, brs, N(CH_2_)_2_), 6.60 (1H, d, *J* = 7.9, H6), 7.49 (1H, d, *J* = 7.9, H5), 7.64 (1H, s, H3) ppm.

#### 3.2.14. Synthesis of 3-(3-Methyl-4-(piperazin-1-yl)phenyl)-5-(trifluoromethyl)-1,2,4-oxadiazole 34

To a solution of **33** (1 mmol) in EtOH, concentrated HCl (3 mL) was added. The reaction mixture was refluxed for 4 h. The mixture was dissolved in water, neutralized with saturated aq. NaHCO_3_, and extracted with EtOAc (3 times). The combined organics were dried (Na_2_SO_4_) and concentrated in vacuo. The residue was purified by column chromatography (eluent CHCl_3_:MeOH = 10:1), Rf = 0.55. White solid, yield 65%, m.p. 78–80 °C. MS (EI), *m*/*z* (*I_relat._*(%)): 312 [M]^+^ (52). Calc. 312.2903, C_14_H_15_F_3_N_4_O. ^1^H NMR (DMSO-d_6_): *δ* 1.87 (1H, s, NH), 2.23 (3H, s, CH_3_Ph), 2.82 (4H, brs, N(CH_2_)_2_), 3.08 (4H, brs, N(CH_2_)_2_), 7.00 (1H, d, *J* = 7.9, H6), 7.43 (1H, d, *J* = 7.9, H5), 7.56 (1H, s, H3) ppm.

#### 3.2.15. General Procedure for the Synthesis of Compounds **35a**,**b**

To a solution of **34** (1 mol) and DMAP (1.3 mol) in DCM, the corresponding acid (1 mol) was added in one portion, followed by the addition of EDCI (1.3 mol) in one portion at rt. The reaction mixture was stirred at rt overnight, after which it was washed successively with 3% aq. HCl and water (3 times). The organic layer was dried and concentrated in vacuo. The residue was triturated with water to provide the product as solid and recrystallized from ethanol.

*(4-(2-Methyl-4-(5-(trifluoromethyl)-1,2,4-oxadiazol-3-yl)phenyl)piperazin-1-yl)(3-phenylisoxazol--5-yl)methanone***35a**, White solid, yield 44%, m.p. 134–136 °C. MS (EI), *m*/*z* (*I_relat._*(%)): 483 [M]^+^ (52). Calc. 483.4425, C_24_H_20_F_3_N_5_O_3_. ^1^H-NMR (DMSO-*d*_6_): *δ* 2.39 (3H, s, CH_3_Ph), 3.05 (4H, m, N(CH_2_)_2_), 3.84 (4H, brt, CON(CH_2_)_2_), 4.37 (2H, brs, NCH_2_), 7.24 (1H, д, *J* = 8.5, H6), 7.55 (3H, m, m, p-Ph), 7.59 (1H, s, isoxazole), 7.86 (1H, d, *J* = 8.5, H5), 7.87 (1H, s, H3), 7.96 (2H, m, o-Ph) ppm. ^13^C-NMR (DMSO-*d*_6_): *δ* 17.68, 46.67, 46.67, 50.24, 50.24, 100.78, 115.90 (q, *J* = 273.2), 118.51, 120.05, 127.10, 127.10, 127.32, 128.17, 128.70, 128.75, 129.71, 129.71, 130.45, 150.10, 155.66, 159.67, 162.39, 166.10 (q, *J* = 43.3), 167.93 ppm.

*(5-Methyl-3-phenylisoxazol-4-yl)(4-(2-methyl-4-(5-(trifluoromethyl)-1,2,4-oxadiazol-3-yl)phenyl)pi-perazin-1-yl)methanone***35b**, White solid, yield 38%, m.p. 119–121 °C. MS (EI), *m*/*z* (I_relat._(%)): 497 [M]^+^ (64). Calc. 497.4691, C_25_H_22_F_3_N_5_O_3_. ^1^H-NMR (CDCl_3_): δ 2.39 (3H, s, CH_3_Ph), 2.57 (3H, s, CH_3_), 2.87–4.07 (8H, m, N(CH_2_)_2_), 6.92 (1H, d, *J* = 7.86, H6), 7.51 (3H, m, m, p-Ph), 7.52 (1H, s, isoxazole), 7.70 (2H, m, o-Ph), 7.92 (1H, d, *J* = 8.5, H5), 7.94 (1H, s, H3) ppm. ^13^C-NMR (DMSO-d_6_): δ 12.40, 17.94, 46.75, 46.75, 50.32, 50.32, 112.0, 115.70 (q, *J* = 273.4), 118.43, 120.00, 127.12, 128.42, 128.65, 128.89, 129.72, 129.72, 130.00, 130.00, 130.67, 149.74, 161.87, 164.00, 166.02 (q, *J* = 43.4), 167.31, 169.98 ppm.

#### 3.2.16. Synthesis of 3-(3-Methyl-4-(4-(prop-2-yn-1-yl)piperazin-1-yl)phenyl)-5-(trifluoromethyl)-1,2,4-oxa-*diazole* **37**

A mixture of **34** (1 mmol), propargyl bromide (1.3 mmol), finely divided K_2_CO_3_ (3 mmol), and KI (0.1 mmol) in acetonitrile was heated at 50 °C for 2 h. The hot mixture was filtered, and the remaining solids were washed with acetonitrile. The combined organic filtrates were concentrated in vacuo. The residue was triturated with water, and precipitate was collected. Yellow solid, yield 77%, m.p. 75–78 °C. MS (EI), *m*/*z* (*I_relat._*(%)): 350 [M]^+^ (71). Calc. 350.3383, C_17_H_17_F_3_N_4_O. ^1^H-NMR (DMSO-*d*_6_): *δ* 2.23 (3H, s, CH_3_Ph), 2.54 (4H, brs, N(CH_2_)_2_), 2.79 (1H, s, CHCCH_2_), 3.12 (4H, brs, N(CH_2_)_2_), 3.33 (2H, s, NCH_2_),7.00 (1H, d, *J* = 7.9, H6), 7.43 (1H, d, *J* = 7.9, H5), 7.56 (1H, s, H3) ppm.

### 3.3. Antiviral Testing of the Compounds

#### 3.3.1. Viruses and Cells

Influenza A virus (strain A/Puerto Rico/8/1934 H1N1) and coxsackievirus 3 (strain Nancy) were obtained from the collection of viruses of the Pasteur Institute (St. Petersburg, Russia). Prior to the experiment, influenza A virus (IAV) and coxsackievirus 3 (CVB3) were grown in MDCK (ATCC # PTA-6500) and Vero cells (ATCC #CCL-81), respectively, for three days at 37 °C and 5% CO_2_. Infectious titers of IAV and CVB3 (in TCID_50_) were determined in MDCK and Vero cells, respectively, by endpoint dilution assay using the following procedure. Cells were seeded into 96-wells plates in Eagles minimal essential medium (MEM) supplemented with 10% fetal bovine serum (FBS). After 24 h, the media was aspirated, the wells were washed with saline, and serial tenfold dilutions of virus stock were added (100 µL per well) in duplicates. The plates were incubated at +4 °C for 1 h, then unbounded virus was discarded, and fresh MEM without FBS was added to the wells (200 µL per well). The plates were incubated at 37 °C in 5% CO_2_ and observed daily for cytopathic effect (CPE). After 72 h, the viral titer was calculated in TCID_50_ using the method of Reed and Muench.

#### 3.3.2. Cytotoxicity Assay

The microtetrazolium test (MTT) was used to study the cytotoxicity of the compounds [24]. The experiment was repeated three times. Vero cells were seeded in 96-well plates in Eagles minimal essential medium (MEM) supplemented with 10% FBS. After 24 h, the media was removed, and the wells were washed with saline. Compounds were dissolved in DMSO, and a series of three-fold dilutions of each compound and pleconaril (1000-4 µg/mL) in MEM without FBS were prepared and added to the cells in quadriplicates (200 µL per well). The maximal concentration of DMSO was 0.5% MEM with 0.5% DMSO and was added to cell control wells. Cells were incubated for 24 h at 37 °C in 5% CO_2_ in the presence of the dissolved compounds. The cells were then washed with saline, and a solution of 3-(4,5-dimethylthiazolyl-2) 2,5-diphenyltetrazolium bromide (ICN Biochemicals Inc., Aurora, OH, USA) (0.5 µg/mL) in MEM was added to the wells (100 µL per well). After 2 h of incubation at 37 °C in 5% CO_2_, the supernatant from wells was discarded, and the formazan residue was dissolved in DMSO (100 µL per well). The optical density of cells was then measured on a Victor 21,440 multifunctional reader (Perkin Elmer, Turku, Finland) at a wavelength of 535 nm and plotted against the concentration of the compounds to generate the dose–response curve. The 50% cytotoxic dose (CC_50_) of each compound (i.e., the compound concentration that causes the death of 50% of cells in a culture, or decreases the optical density twice as compared to the control wells) was calculated using four-parameter logistic nonlinear regression model. For some compounds (**35a** and **35b**) cytotoxicity towards MDCK cell line was determined using the procedure above, but the cells were exposed to compounds for 72 h.

#### 3.3.3. Antiviral Activity Determination

Antiviral activity of the compound against CVB3 was evaluated using viral yield reduction assay. The experiment was repeated three times. Vero cells were seeded in MEM supplemented with 5% FBS in 24-well plates. When the cells confluence reached 100%, giving an approximate cell density of 0.2 × 10^6^ per well, the compounds were dissolved in DMSO, and a series of three-fold non-toxic dilutions of each compound (600–6 µg/mL) and pleconaril (600–0.6 µg/mL) in MEM without FBS was prepared, added to the cells (500 µL per well), and incubated at 37 °C in 5% CO_2_. After 1 h, viral suspension in MEM without FBS was added to the all wells at MOI 0.01 (500 µL per well) except cell control, and the plates were incubated at 4 °C for 1 h. Thereafter, the cell supernatant was removed, and MEM without FBS was added to all wells (1 mL). After 24 h of incubation at 37 °C in 5% CO_2_, the viral progeny infectious titers (in TCID_50_) for each compound concentration, cell control, and virus control wells were determined in Vero cells by endpoint dilution assay. The supernatants from corresponding wells of 24-plates were serially diluted in titer tubes and added to 96-well plates in duplicates (200 µL per well). The plates were incubated at 37 °C in 5% CO_2_ and observed daily for cytopathic effect. After 72 h, the viral titer in each compound concentration, cell control, and virus control wells was calculated in TCID_50_ using the method of Reed and Muench. The infectious titer of virus progeny was plotted against the concentration of the compounds to generate the dose–response curve. The 50% inhibition concentration (IC_50_) of each compound tested (i.e., the compound concentration that decreases the infectious viral progeny titer twice as compared to the control wells) was calculated using four-parameter logistic nonlinear regression model. Selectivity index (SI) was calculated for each compound tested as a ratio of CC_50_ to IC_50_ values.

Antiviral activity of the compound against IAV was evaluated using hemagglutination test. The experiment was repeated three times. MDCK cells were seeded in MEM supplemented with 5% FBS in 96-well plates. When the cells confluence reached 100%, the plate was washed with saline, the tested compounds were dissolved in DMSO, and a series of three-fold non-toxic dilutions of each compound (600–6 µg/mL) in MEM without FBS was prepared and added to the cells (100 µL per well). Tenfold dilutions of viral suspension in MEM without FBS were added to all wells (100 µL per well) except cell control, and the plates were incubated at 37 °C for 72 h. Thereafter, the cell supernatant (100 µL) was transferred to a “V bottom” 96-well microtiter plate and mixed with 100 µL of 1% chicken erythrocytes (RBC). After 1 h, cell control was checked for complete settling of RBCs, and the viral titer was determined. The 50% inhibitory concentration (IC_50_) of each compound tested (i.e., the compound concentration that decreases the viral titer twice as compared to the control wells) was calculated using four-parameter logistic nonlinear regression model. Selectivity index (SI) was calculated for each compound tested as a ratio of CC_50_ to IC_50_ values.

## 4. Conclusions

We have synthesized a series of novel pleconaril-based compounds with modified *O*-alkyl linker. All the derivatives were characterized by their MS and NMR data. Synthesized target compounds were evaluated for their in vitro antiviral activity against coxsackievirus B3 strain Nancy. Among these compounds, **21** with an IC_50_ value of 6.8 μM and SI of 248, is the most active anticoxsackievirus agent compared to other studied compounds (including pleconaril) with low cytotoxicity. The results of this study demonstrate the possibility to further improve pleconaril and evolve it to develop novel potent selective anti-coxsackievirus inhibitors that have activity against the B3 Nancy strain.

## Figures and Tables

**Table 1:**
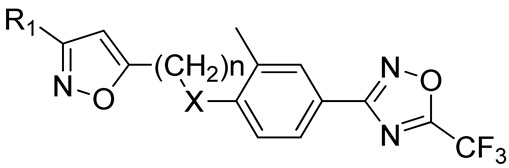
Antiviral activity of synthesized compounds towards coxsackievirus B3 strain Nancy.

№	R_1_	X	*n*	CC_50_, μM	IC_50_ towards CVB3, μM	SI
6a	COOEt	S	3	484.8 ± 20.1	21.0 ± 1.5	23
6b	Me	S	3	>2608.3	15.6 ± 1.4	>167
7	CONMe_2_	S	3	>2270.45	18.6 ± 1.3	>122
12a	COOEt	COO	2	682.8 ± 30.4	9.1 ± 0.8	75
12b	Me	COO	2	464.2 ± 30.3	13.1 ± 0.9	35
20a	COOEt	CONH	2	1724.6 ± 70.5	18.2 ± 1.6	95
20b	Me	CONH	2	1743.2 ± 120.2	76.2 ± 5.3	23
21	CONMe_2_	CONH	2	1689.6 ± 70.7	6.8	248
28a	CONMe_2_	piperazine	0	1867.1 ± 90.4	9.1 ± 0.7	205
28b	Me	piperazine	1	2455.2 ± 170.5	41.7 ± 2.1	59
28c	COOEt	piperazine	0	53.2 ± 4.5	48.7 ± 4.4	1
28d	COOMe	piperazine	1	203.8 ± 14.7	3.9 ± 0.3	52
35a	3-phenylisoxazole-5-carbonyl	piperazine	0	1522.4 ± 70.3	>620.5	<2
35b	5-methyl-3-phenylisoxazole-4-carbonyl	piperazine	0	2010.1 ± 120.8	16.1 ± 1.1	125
Pleconaril				2396.7 ± 160.2	18.4 ± 1.5	130

**Table 2:** Antiviral activity of compounds **35a,b** towards influenza virus A/Puerto Rico/8/34 (H1N1).

№	CC_50_, μM	IC_50_ Towards IAV, μM	SI
35a	1449.3 ± 102.1	>492.8	<3
35b	>603.6	>603.6	n/a ^1^

^1^ n/a—not applicable.
